# Attentiveness and mental health in adolescents with moderate-to-severe atopic dermatitis without ADHD

**DOI:** 10.1007/s00403-024-03210-x

**Published:** 2024-07-30

**Authors:** Amy S. Paller, Mercedes E. Gonzalez, Sarah Barnum, Judith Jaeger, Liyang Shao, Zafer E. Ozturk, Andrew Korotzer

**Affiliations:** 1 Northwestern University Feinberg School of Medicine, Chicago, IL 60611 USA; 2 Pediatric Skin Research, Miami, FL 33146 USA; 3 CognitionMetrics, LLC, Stamford, CT 06903 USA; 4 Regeneron Pharmaceuticals Inc., Tarrytown, NY 410591-6717 USA; 5 Sanofi, Cambridge, MA 02142 USA

**Keywords:** Atopic dermatitis, Pruritus, ADHD, Conners, Continuous Performance Test, Attention, Mental health

## Abstract

Patients with moderate-to-severe atopic dermatitis (AD) experience intense chronic itch and impaired sleep. Reports from parents and teachers suggest that AD patients may also have attention problems. However, attention has not yet been directly assessed in AD patients. We utilized an objective, computer-based continuous performance test (CPT) validated for use in attention-deficit/hyperactivity disorder (ADHD) diagnosis to formally evaluate attention in adolescent AD subjects. This was a single-visit, cross-sectional, non-interventional study of moderate-to-severe (Investigator’s Global Assessment [IGA] ≥ 3) AD subjects aged 12–17 years without clinician-diagnosed ADHD. Attention was evaluated using two performance-based measures: Conners, CPT-3 and the Stroop Color and Word Test. The primary parameter was CPT-3 detectability (d’) measure. Lesional severity measures included Eczema Area and Severity Index (EASI) and body surface area (BSA) involvement. Subjects completed self-report rating scales assessing sensory responsiveness patterns (Adult/Adolescent Sensory Profile [AASP]), itch (Peak Pruritus Numerical Rating Scale [PP-NRS]), skin pain, quality of life, sleep, anxiety, and depressive symptoms. A total of 44 subjects were included in the study (61.4% female; mean age 15.0 [SD 1.78] years; mean EASI 20.4 [SD 7.8]; mean PP-NRS 7.0 [SD 1.8]). Results indicated substantial disease impact on sleep, quality of life, and comorbid anxiety and depressive symptoms. The mean (SD) Conners, CPT-3 dʹ T-score was 48.7 (SD 10.7), similar to the expected mean from a randomly selected age/gender-matched sample of the general population (50 [SD 10], by definition). Overall, 13.6% of subjects exhibited a dʹ T-score ≥ 60 (clinically significant poor performance), which was not greater than the expected general population value (15.9%). Subject-level data review by two psychologists determined that only 2 subjects demonstrated an overall response pattern that clearly indicated attention deficit. Many subjects had atypical sensory responsiveness profiles: sensory hypersensitivity (38.6%), sensory avoidance (50%), and low registration (hypo-sensitivity, 36.4%). Adolescents with moderate-to-severe AD without existing ADHD diagnosis did not demonstrate greater attention problems on performance-based measures than would be expected in age/gender-matched peers.

**Trial registration** NCT05203380.

**Supplementary Information:**

The online version contains supplementary material available at 10.1007/s00403-024-03210-x.

## Plain Language Summary

Atopic dermatitis (often shortened to AD) is a long-term skin disease that causes intense itching. It affects patients’ lives in many ways, including interrupting their sleep. Parents and teachers of young people with AD have sometimes suggested that AD may also cause attention problems. But this has never been tested properly.

We measured the attention of 44 adolescents aged between 12 and 17 years who all had moderate-to-severe AD. We used computerized tests of attention that were developed for young people with ADHD (attention deficit hyperactivity disorder). Also, we made sure that none of the 44 patients had also been diagnosed with ADHD. The severity and extent of the patients’ AD was measured by doctors. We also used some measures that allowed the patients to report how AD affected their lives, including things like itch, skin pain, quality of life, sleep, anxiety, and depression.

The adolescent patients reported that AD had a negative effect on various areas of their lives, including sleep and quality of life, and that it resulted in anxiety and symptoms of depression. However, the results of the attention tests in adolescents with AD were similar to what would usually be expected in adolescents without AD. Only 2 of the 44 patients with AD were found to have clear evidence of attention problems.

The study concluded that adolescents with moderate-to-severe AD did not have any greater attention problems than would usually be expected in adolescents without AD.

**Supplementary Information:**

The online version contains supplementary material available at 10.1007/s00403-024-03210-x.

## Introduction

Atopic dermatitis (AD) is a chronic, systemic, inflammatory disease characterized by intense itch [1] and sleep loss [2–5]. AD exerts a substantial psychological burden; symptoms of depression and anxiety are commonly reported in patients with moderate-to-severe AD [6–8], and young children (aged 18–48 months) with AD exhibit greater clinginess and fearfulness than matched controls [9]. Since patients in all age groups with moderate-to-severe AD may spend up to 4 months annually experiencing an eczematous flare [10], the psychological impact of AD can be chronic and transcend periods of disease quiescence. Adults with moderate-to-severe AD report worsened mental health compared with the overall US population [11].

Itch is an inherently strong consumer of attention [12], and sleep loss is likewise associated with inattentiveness [13], suggesting that inattentiveness could be part of the overall psychological burden of AD. Several studies have used subjective parent- and/or teacher-rated assessment tools to attempt to address the potential burden of inattentiveness in children with versus without AD (and without diagnosed ADHD). One study demonstrated greater subjective reports of inattentiveness in children with AD [14], while a second study showed that children with AD had a significantly higher prevalence of observer-reported ADHD symptoms [15], despite exclusion of patients with an ADHD diagnosis. Another study concluded that severe AD in pre-school age children was correlated with poor sleep health and attention dysregulation [16]. These findings parallel results observed in a study of children and adolescents with chronic pain, of whom 20% manifested subjectively rated, clinically significant ADHD symptoms, despite fewer than 5% having been diagnosed with ADHD [17].

This study was designed to assess whether adolescents with moderate-to-severe AD demonstrate impaired ability to sustain attention using an objective task. Conners, Continuous Performance Test, Third Edition (CPT-3), was chosen as the primary outcome assessment tool due to its frequent use in clinical evaluation of attention problems and the correlation between outcome parameters of CPT-3 and the specific symptoms of ADHD, including those suggested by the study in AD patients by Kruse and colleagues [14, 18]. Specifically, Conners, CPT-3 yields a set of parameters that describe the subject’s performance, which a neuropsychologist can use to evaluate four distinct domains of attention: inattentiveness, impulsivity, sustained attention, and vigilance. CPT-3 can facilitate ADHD diagnosis, in that ADHD patients exhibit worse performance across most parameters of the task than the general population; patients with other disorders with impaired executive function, such as anxiety disorder and autism spectrum disorder, also demonstrate worse performance on Conners, CPT-3. Conners, CPT-3 has been validated for use in adolescent populations [19].

### Subjects

This single-visit, cross-sectional, non-interventional study enrolled adolescents aged ≥ 12 to ≤ 17 years with moderate-to-severe AD, defined by having an Investigator’s Global Assessment (IGA) score of 3 or 4 [20], an Eczema Area and Severity Index (EASI) score of ≥ 12 [21], a Peak Pruritus Numerical Rating Scale (PP-NRS) score of ≥ 4 [22], and Body Surface Area (BSA) of AD involvement of ≥ 10% at enrollment [23].

Exclusion criteria included use of dupilumab within 6 months, systemic antihistamine or nicotine use within one week, or use of the following ADHD medications within 8 weeks or 5 half-lives (whichever was longer) prior to the visit: methylphenidate, dexmethylphenidate, serdexmethylphenidate, amphetamine, dextroamphetamine, lisdexamfetamine, guanfacine, atomoxetine, clonidine, and viloxazine. Also excluded were subjects with a history of clinician-diagnosed ADHD, autism spectrum disorder, epilepsy, major depressive disorder, mania or bipolar disorder, or any Diagnostic and Statistical Manual-V (DSM-V) psychotic disorder such as schizophrenia. Other exclusion criteria were substance abuse, including alcohol or nicotine, in the prior 2 years or requiring institutionalization.

### Subject-reported assessments

AD symptoms were quantified by the PP-NRS and Skin Pain (SP)-NRS [24]. Other measures included the Children’s Dermatology Life Quality Index (CDLQI) [25], Patient-Reported Outcomes Measurement Information System (PROMIS) Sleep Disturbance [26], and Hospital Anxiety and Depression Scale (HADS) [27], which were used to assess quality of life affected by AD, sleep disturbance, and symptoms of anxiety and depression, respectively.

Sensory responsiveness patterns were assessed using the Adult/Adolescent Sensory Profile (AASP), a 60-item self-reported questionnaire that measures sensory responsiveness patterns in six sensory modalities, including taste/smell, movement, vision, touch, activity, and auditory processing [28]. Four domains of sensory processing were assessed separately by 15 questions each: sensory sensitivity, low registration (hypo-sensitivity), sensory avoidance, and sensory seeking; for each domain, normed total scores ranged from 15 to 75. For adolescents (aged 11–17 years), a subject was designated “similar to most people” if they had scores of 26–40 for sensory sensitivity and sensory avoidance, 27–40 for low registration, and 42–58 for sensory seeking.

### Neuropsychologic assessments

The primary assessment tool was Conners, CPT-3, an objective and computerized measure of attention-related problems [19]. CPT-3 is a 14-minute computerized task, in which the subject is shown strings of letters in 6 blocks and at inter-stimulus intervals (ISIs) of 1, 2, or 4 seconds. The subject is asked to press a specific key as quickly as possible when each letter is displayed, but to not respond when the letter “X” appears on the screen. Response accuracy and response times are measured and converted to standard scores (T-scores) using age and gender norms. Detectability (d’), which measures the subject’s ability to discriminate “non-targets” (i.e., the letter X) from “targets” (all other letters), was used as the primary outcome parameter. Additional norm-referenced data from this task included response style, error analysis (omission errors, commission errors, perseverations), reaction time statistics (hit reaction time [HRT]), HRT standard deviation, variability, HRT block change, and HRT inter-stimulus interval), as well as raw score omissions and commissions by block.

The Stroop Color and Word Test was used to measure selective attention, or the subject’s ability to attend to a salient stimulus while inhibiting an obtrusive irrelevant stimulus [29, 30]. The methods of the Stroop Color and Word Test are detailed in the supplementary methods (Supplementary appendix S1). By definition, average ability to selectively attend to the salient stimulus is represented by a Stroop Interference T-score of 50, with higher values representing better performance (Supplementary appendix S1).

All subject-level data from Conners, CPT-3 and the Stroop tests were interpreted independently by two clinical psychologists (SB and JJ), who categorized subjects into three groups: Group 1: very consistent with impaired attention; Group 2: possible evidence of impaired attention, and Group 3: no evident impairment.

### Sample size

By definition, the mean dʹ T-score of a randomly selected sample is expected to be 50, with a standard deviation (SD) of 10; equivalently, 15.9% of a random sample would be expected to have a dʹ T-score of at least 60, a threshold that is considered to represent clinically significant poor performance (here, higher values for T-scores represent worse performance). We hypothesized that a modest but measurable degree of inattentiveness would be prevalent in adolescents with moderate-to-severe AD. To determine an adequate sample size, we therefore assumed that this population would have mean a dʹ T-score of 55 and SD of 10; equivalently, 30.9% of the subjects with AD were expected to have a dʹ T-score of ≥ 60. Under these assumptions, it was determined that 44 subjects would need to be enrolled to test the hypothesis that there are higher rates of attention problems in the AD sample.

### Ethical conduct of the study

This study was conducted in accordance with consensus ethical principles derived from international guidelines, including the Declaration of Helsinki, and consistent with the International Conference on Harmonisation Good Clinical Practice guideline and applicable regulatory requirements. All subjects provided informed assent and a parent or caregiver provided informed consent prior to the patient undergoing any study-related procedure. This study was registered at ClinicalTrials.gov (Identifier: NCT05203380).

## Results

### Patient demographics

A total of 45 subjects were enrolled at 9 US sites between January 2022 and March 2023; however, one individual with a low PP-NRS score was enrolled in error and excluded from further analysis. The evaluable population (*N* = 44) was 61.4% female and had a mean (SD) age of 15.0 (1.78) years (Table 1). Concomitant medical conditions included seasonal allergy (40.9%), food allergy (34.1%), asthma (31.8%), allergic rhinitis (18.2%), and acne (11.4%) (Table 1). Use of at least one medication was reported by 35 (79.5%) subjects, most frequently topical corticosteroids (20 subjects; 45.5%) and drugs for airway diseases (13; 29.5%). Four subjects used immunosuppressants (4; 9.1%).

**Table 1:** Patient baseline demographics

Efficacy analysis set	(*n* = 44)
Age, mean (SD)	15.0 (1.78)
Female, *n*(%)	27 (61.4)
Race, *n* (%)	
White	28 (63.6)
Black/African American	8 (18.2)
Asian	6 (13.6)
Multiple	2 (4.5)
Ethnicity, *n*(%)	
Hispanic/Latino	29 (65.9)
Not Hispanic/Latino	15 (34.1)
Concomitant medical conditions^a^	
Seasonal allergy	18 (40.9%)
Food allergy	15 (34.1%)
Asthma	14 (31.8%)
Allergic rhinitis	8 (18.2%)
Acne	5 (11.4%)

^a^Concomitant medical condition reported in ≥ 10% of subjects

A total of 34 (77.3%) subjects had IGA = 3 (moderate) and 10 (22.7%) had IGA = 4 (severe) AD. Subjects had mean (SD) EASI scores of 20.4 (7.8), BSA of 33.8 (17.9), and mean (SD) symptom scores of 7.0 (1.8) for PP-NRS and 5.2 (2.9) for SP-NRS. Together, these scores are consistent with poorly controlled moderate-to-severe AD at time of visit. Subjects had mean scores (SD) of 11.3 (7.4) for CDLQI, 61.1 (8.9) for PROMIS Sleep Disturbance, 10.3 (2.3) for HADS Anxiety, and 13.0 (2.0) for HADS Depression, indicating considerable quality-of-life and sleep impairment, in addition to high levels of anxiety and depressive symptoms, respectively (Table 2)

**Table 2:** Patient baseline disease characteristics

Disease characteristic	(*n* = 44)
PROMIS Sleep Disturbance (T-score), mean (SD)	61.1 (8.9)
IGA = 3, *n* (%)	34 (77.3)
BSA (percentage range: 0–100%), mean (SD)	33.8 (17.9)
EASI (score range: 0–72), mean (SD)	20.4 (7.8)
HADS Depression (score range: 0–21), mean (SD)	13.0 (2.0)
CDLQI (score range: 0–30), mean (SD)	11.3 (7.4)
HADS Anxiety (score range: 0–21), mean (SD)	10.3 (2.3)
Peak Pruritus NRS (score range: 0–10), mean (SD)	7.0 (1.8)
Skin Pain NRS (score range: 0–10), mean (SD)	5.3 (2.9)

Mean (SD) scores for each of the 4 domains of the AASP appeared to be close to normal: sensory sensitivity 38.0 (10.5), sensory avoidance 41.3 (10.6), low registration 37.4 (9.6), and sensory seeking 45.3 (7.9). However, a substantial proportion of the subjects had values that were more or much more than most people for the domains of sensory sensitivity (17 of 44 [38.6%]), sensory avoidance (22 of 44 [50%]), and low registration (16 of 44 [36.4%]). In contrast, only 2 subjects (4.5%) had atypically high sensory seeking values.

### Neuropsychologic outcomes

In our sample, the mean (SD) Conners, CPT-3 dʹ T-score was 48.7 (10.7), and 13.6% subjects (CI 3.5–23.8%) had a dʹ T-score ≥ 60, consistent with the proportion expected in the general population. Stroop interference T-scores were not calculated for 7 subjects, per the procedure described in Supplemental Methods. For the remaining 37 subjects, the mean (SD) interference T-score was 55.5 (7.54); the median interference T-score was 55 (IQR 52–61).

Further review of the CPT-3 and Stroop data was conducted by two psychologists, who independently reviewed the results for all parameters of these tests for each participant. Independent ratings had high levels of agreement, suggesting consistency of interpretation across clinicians. Both psychologists agreed that the CPT-3 results for one subject were invalid and excluded this patient from their review. Of the 43 valid CPT-3 profiles, 5 subjects (11.6%) had a dʹ T-score ≥ 60, which remains numerically lower than the expected proportion (15.9%) of individuals of this age group. Regarding the Stroop Interference scores, 2 subjects were rated as having a “below average” performance, while 12 were rated as having either “above average” or “very good” performance (the remaining subjects with valid interference scores had average performance).

Given the Conners, and Stroop subject-level data, the psychologists categorized 2 of 43 subjects (4.7%) into Group 1 (clear indication of attention problems) and 9 of 43 (20.9%) into Group 2 (possible evidence of attention problems). Of the subjects categorized as Group 1, the Stroop Interference T-score of one subject was normal (57), and one subject’s score was invalid. Given the low numbers of subjects with clear evidence of attention problems, the distribution of AD lesional or symptomatic scores across these three groupings could not be compared.

### Correlations of neuropsychologic outcomes and sensory processing with AD signs and symptoms

No significant correlations were noted between Conners, CPT-3 d’ T-score and any measure of AD signs and symptoms, sleep disturbance, quality of life, or mood (Table 3). Similarly, no correlations were noted between Stroop Interference T-score and any of the same measures (not shown).

**Table 3:** Correlations of Conners, CPT-3 d’ T-score and sensory sensitivity and avoidance scores, with measures of AD signs, symptoms, and disease burden (*N* = 44)

		d’ T-score	Sensory sensitivity	Sensory avoidance	Low registration
EASI	r^a^	−0.190	−0.287	−0.225	−0.226
	*p-*value	0.219	0.059	0.143	0.141
BSA	r^a^	−0.194	−0.265	−0.246	−0.234
	*p-*value	0.209	0.083	0.108	0.126
PP-NRS	r^a^	0.051	0.274	0.436	0.318
	*p-*value	0.746	0.072	**0.003**	**0.035**
SP-NRS	r^a^	0.030	0.294	0.367	0.233
	*p-*value	0.848	0.052	**0.014**	0.128
PROMIS Sleep Disturbance	r^a^	−0.132	0.584	0.529	0.486
	*p-*value	0.397	**< 0.0001**	**0.0002**	**0.0007**
CDLQI	r^a^	0.002	0.423	0.599	0.382
	*p-*value	0.991	**0.004**	**< 0.0001**	**0.010**
HADS Anxiety subscale	r^a^	0.167	0.5778	0.639	0.473
	*p-*value	0.281	**< 0.0001**	**< 0.0001**	**0.001**
HADS Depression subscale	r^a^	−0.087	−0.211	−0.076	−0.075
	*p-*value	0.577	0.171	0.628	0.633
IGA	r^a^	−0.122	−0.209	−0.088	−0.155
	*p-*value	0.433	0.175	0.573	0.318

^a^ r, Pearson correlation coefficient. Sensory seeking scores did not significantly correlate with any of the parameters listed in this table

Significant correlations were observed for the sensory sensitivity score and PROMIS Sleep Disturbance, CDLQI, and the HADS Anxiety subscale. Significant correlations were also observed for the sensory avoidance score and Peak Pruritus NRS, Skin Pain NRS, PROMIS Sleep Disturbance, CDLQI, and the HADS Anxiety subscale (Table 3). Interestingly, there was a trend towards an inverse correlation between the sensory sensitivity score and lesional signs (EASI score, BSA, ***p-***values < 0.1; Table 3). Low registration scores correlated with Peak Pruritus-NRS, PROMIS Sleep Disturbance, CDLQI, and HADS Anxiety subscale. (Table 3). Sensory seeking did not correlate with any of the parameters listed in Table 3 (data not shown).

## Discussion

In this cross-sectional, non-interventional study, adolescents with moderate-to-severe AD (without clinician-diagnosed ADHD) had no reduced ability to sustain attention on an objective, performance-based continuous task, or in their ability to maintain selective attention. Specifically, the Conners, CPT-3 task has high sensitivity to detect problems in inattentiveness, impulsivity, sustained attention, and vigilance [19]. The Stroop Color and Word Test measures selective attention [29], or the ability to attend to a salient stimulus when confronted with an obtrusive irrelevant stimulus. The combined results from these two neuropsychologic assessment tools demonstrate that the subjects in our sample did not differ from what would be expected from age/gender-matched subjects in the general population across any of these dimensions of attention. This suggests that teens with moderate-to-severe AD are capable of high-quality attention to task and do not have underlying attention deficits, at least for fixed periods of time in a controlled setting.

Our inclusion criteria were meant to ensure enrollment of subjects with moderate-to-severe AD uncontrolled by prescription topical medications (i.e., candidates for escalation of care) [31]. The subjects in our sample clearly suffered from high disease burden, with an average BSA of 33.8%. Their itch scores, mood, and anxiety symptoms, and the quality-of-life impact of AD were similar to those of adolescents with moderate-to-severe AD recruited into a recent trial of a systemic biologic (Table S1) [32].

Subjects were also assessed for their sensory responsiveness, using an instrument (the Adult/Adolescent Sensory Profile [AASP]) that quantifies self-reported “thresholds” of sensory stimuli (either sensory sensitivity or its opposite, low registration) and behavioral responses to stimuli (either sensory avoidance or sensory seeking) across multiple sensory modalities. A substantial proportion of subjects in our sample were rated as having atypically high sensory sensitivity (38.6%), low registration (36.4%), and sensory avoidance (50%), but only 2 (less than 5%) rated high for sensory seeking.

Scores for sensory sensitivity, low registration, and sensory avoidance correlated highly with sleep impairment, quality of life impairment, and anxiety. Interestingly, low registration but not sensory sensitivity correlated significantly with degree of itch.

Many items in the sensitivity and avoidance domains emphasize distractibility and subject behaviors meant to cope with such distractibility. As examples, for sensory sensitivity: “I am distracted if there is a lot of noise around,” and “I am bothered by unsteady or fast-moving visual images in movies or TV,” and for sensory avoidance: “I stay away from noisy settings” and “I only eat familiar foods.” It is notable that the AASP surveys distractive stimuli across sensory modalities (not only tactile stimuli), since the predominant symptom of AD is chronic itch.

We did not objectively measure sensory thresholds or distractibility in our sample; it is possible that respondents overstated the degree to which they actually experience sensory integration difficulties, due to subjective reporting bias. If we take their self-reports at face value, we do not know whether sensory sensitivity would have affected their performance on the CPT task, which was done in conditions of minimal ambient distractions. Sensory distractions can affect performance of sustained attention tasks in various psychiatric clinical populations, such as adolescent patients with ADHD [33, 34] and adults with schizophrenia [35]. However, non-clinical samples of adults and adolescents can perform such tasks with little or no decrement in the presence of sensory distractors [34, 35]. Indeed, by adolescence, selective attention abilities appear to be fully formed, and distractor interference effects in such tasks may be related to issues around sensory perception [36, 37].

It would be of interest in future research to determine whether neurotypical adolescents with moderate-to-severe AD, with demonstrably average attention abilities, are nevertheless more susceptible to distraction interference effects in attention tasks than healthy controls, for at least two reasons. First, such distractor interference effects in adolescents have been shown to be a predictor of distractibility in the classroom [36]. But secondly, there is evidence that sensory stimuli (but not a cognitive attention task) can distract from itch, making the role of sensory processing directly relevant to AD patients. For example, Leibovici and others [38] observed that chronic itch patients had reduced scratching and a lower itch rating when exposed to distracting audiovisual stimuli. In contrast, Stumpf and others [39] performed functional magnetic resonance imaging on subjects while they performed a Stroop task; the Stroop task was intended to distract the subjects from a pruritic stimulus. The imaging studies confirmed that the performance of the Stroop test activated brain regions known to be associated with attention control; however, the Stroop test failed to distract the subjects from the itch.

The exclusion of subjects with clinician-diagnosed ADHD is an important feature of this study, given that numerous epidemiologic studies have shown an association between ADHD and AD. [40–45]. This entry criterion was established to plausibly associate any findings of poor attentiveness with AD itself (presumably related to poor sleep or distractibility due to intense itch), rather than comorbid ADHD.

Recent findings utilizing parent/teacher-rated scales suggested the presence of ADHD-like symptoms in pediatric AD patients without ADHD [14, 15]; combined with the epidemiological association of AD with ADHD, it is perhaps surprising that attention abilities appeared unimpaired in our sample. However, in the report by Feng and colleagues [15], it was hyperactivity that was significantly higher in AD than control patients; inattention was not different between AD and controls. This is also supported by the report of Ma and colleagues [46] wherein it was determined that in patients with AD without neurodevelopmental comorbidities such as ADHD, there was no significant impairment in cognitive function. In contrast, Kruse [14] reported that AD patients had significantly higher symptoms of inattention. However, some of these symptoms may also relate conceptually to distractibility rather than inattentiveness; for example, “is easily distracted by noises or other stimuli,” and “is forgetful in daily activities.”

Strengths of this study include its evaluation in the dermatology practice setting using computerized methodology of a poorly studied potential feature of adolescents and children with moderate-to-severe AD. Furthermore, this study supports the usefulness of the Conners CPT-3, administered in an outpatient healthcare setting, in distinguishing ADHD from sensory processing concerns or distraction due to itch. Distinguishing these potential associations with AD could provide support for physicians trying make decisions about referral for ADHD or focus on skin-based interventions and coping strategies for patients who are experiencing distracting itch symptoms in real-world settings.

This study has several notable limitations. We excluded subjects who took systemic antihistamines (including non-sedating antihistamines) within a week prior to the study visit. Given that approximately 60% of adolescents with moderate-to-severe AD have allergic rhinitis, which is commonly treated with antihistamines, many potential candidates for study were ineligible [32]. The enrolled sample was 61% female, and females tend to perform better on Conners, CPT-3. However, all analysis was performed using T-scores, which are both age- and gender-adjusted.

A further limitation of this study is a lack of data concerning whether the subjects had behavioral signs (i.e., argumentativeness, anger outbursts) that might be associated with conditions involving poor attentiveness, such as were utilized in previous studies [14, 15]. However, results from the subject-rated assessment tools in this study suggest a sample of adolescents who had psychological concerns that may have led to altered behavior patterns, and some subjects (38.6%) reported being unusually sensitive to sensory stimuli across sensory modalities and avoided sensory stimuli when possible (50%). The latter finding is consistent with previously published findings in children with AD using the AASP [47].

In conclusion, we found that inattentiveness, as measured by CPT-3 in adolescents with moderate-to-severe AD, occurs at a prevalence that would be expected in a population without AD. Future research could further explore the sensory issues of children and adolescents with AD, comparing them to age-matched patients with other pruritic dermatological conditions.

## Electronic supplementary material

Below is the link to the electronic supplementary material.

Supplementary Material 1

Supplementary Material 2

## Data Availability

Data of the present study can be requested from the corresponding author.
