# Supplementary material for: Attentiveness and mental health in adolescents with moderate-to-severe atopic dermatitis without ADHD
Source: Arch Dermatol Res. 2024 Jul 30;316(8):497. doi: 10.1007/s00403-024-03210-x (PMC11288989; doi:10.1007/s00403-024-03210-x)
Supplement: Supplementary file 1 — Supplementary Material 1 [file 403_2024_3210_MOESM1_ESM.pdf]

Attentiveness and mental health in adolescents with moderate to severe atopic dermatitis without ADHD

Amy S. Paller[1], Mercedes E. Gonzalez[2], Sarah Barnum[3], Judith Jaeger[3], Liyang Shao[4], Zafer E. Ozturk[5], Andrew Korotzer[4]

[1] Northwestern University Feinberg School of Medicine, Chicago, IL 60611, USA

[2] Pediatric Skin Research, Miami, FL 33146, USA

[3] CognitionMetrics, LLC. Stamford, CT 06903, USA

[4] Regeneron, Tarrytown, NY 10591-6717, USA

[5] Sanofi, Cambridge, MA 02142, USA

Corresponding author: Amy S. Paller (apaller@nm.org)

Supplementary Methods

Neuropsychologic assessments

The Stroop Color and Word test is made up of three individual timed trials. [**1**,**2**]. In the word trial ("W"), the subject was presented with the words "RED", "GREEN" and "BLUE" arranged randomly and printed in black ink on white paper. They were instructed to read the words on the page as quickly as possible. In the Color trial ("C"), the subject was shown "XXXX" printed in either red, green or black ink and instructed to name the colors as quickly as possible. Finally, in the Color-Word trial ("CW"), subjects were shown the words "RED", "GREEN" and "BLUE" printed in ink of a color that does not match the word (e.g., the word "BLUE" could be printed in red letters); subjects are asked to name the color of the ink the words were printed in – not the word itself.

The number of correctly identified items in 45 seconds during each trial were then calculated (e.g., W, C, and CW). The Color-Word Raw Score (CW) is typically lower than that of the other Raw scores, because of the requirement that the subject ignore the word and name the color; this "interference" is expected to slow the subject's performance. Raw Stroop "Interference scores" were calculated by subtracting the CW from a predicted CW, derived from the first two trials: predicted (CW) = (W * C) / (W + C); [**2**] this score was then converted into a T-score using Table VI of Appendix A of the Stroop Test Manual. [**3**] Score patterns in which the Color-Word Raw Score was higher than the Color Raw Score were considered invalid, and an interference score was not calculated for these subjects.

References

1. Stroop JR (1935) Studies of interference in serial verbal reactions. J Exp Psychol 18:643–662. https://doi.org/10.1037/h0054651
2. Scarpina F, Tagini S (2017) The Stroop Color and Word Test. Front Psychol 8:557. https://doi.org/10.3389/fpsyg.2017.00557
3. Golden CJ, and Freshwater SM (2002) The Stroop Color and Word Test: A Manual for Clinical and Experimental Uses. Chicago, IL: Stoelting.
