# Supplementary material for: Attentiveness and mental health in adolescents with moderate-to-severe atopic dermatitis without ADHD
Source: Arch Dermatol Res. 2024 Jul 30;316(8):497. doi: 10.1007/s00403-024-03210-x (PMC11288989; doi:10.1007/s00403-024-03210-x)
Supplement: Supplementary file 2 — Supplementary Material 2 [file 403_2024_3210_MOESM2_ESM.pdf]

Attentiveness and mental health in adolescents with moderate to severe atopic dermatitis without ADHD

Amy S. Paller[1], Mercedes E. Gonzalez[2], Sarah Barnum[3], Judith Jaeger[3], Liyang Shao[4], Zafer E. Ozturk[5], Andrew Korotzer[4]

[1] Northwestern University Feinberg School of Medicine, Chicago, IL 60611, USA

[2] Pediatric Skin Research, Miami, FL 33146, USA

[3] CognitionMetrics, LLC. Stamford, CT 06903, USA

[4] Regeneron, Tarrytown, NY 10591-6717, USA

[5] Sanofi, Cambridge, MA 02142, USA

Corresponding author: Amy S. Paller (apaller@nm.org)

Regeneron - Internal

Table S1 Comparison of baseline parameters for adolescents with moderate-to-severe AD enrolled in this single-visit, cross-sectional, non-interventional study (NCT05203380), and in a prior randomized dupilumab clinical trial [**1**].

	**R668-AD-1526 (N = 251)**	**NCT05203380 (N = 44)**
**EASI, mean (SD)**	35.5 (14.2)	20.4 (7.8)
**Percent BSA, mean (SD)**	56.5 (23.0)	33.8 (17.9)
**Peak Pruritus NRS, mean (SD)**	7.6 (1.7)	7.0 (1.8)
**CDLQI, median (IQR)**	12.0 (8.0 – 20.0)	12.0 (5.0 – 15.5)
**HADS Anxiety subscale, mean (SD)**	7.8 (4.6)	10.3 (2.3)
**HADS Depression subscale, mean (SD)**	4.6 (4.1)	13.0 (2.0)
**Percent IGA = 3**	46.2	77.3

Reference

1. Simpson EL, Paller AS, Siegfried EC, Boguniewicz M, Sher L, Gooderham MJ, Beck LA, Guttman-Yassky E, Pariser D, Blauvelt A, Weisman J, Lockshin B, Hultsch T, Zhang Q, Kamal MA, Davis JD, Akinlade B, Staudinger H, Hamilton JD, Graham NMH, Pirozzi G, Gadkari A, Eckert L, Stahl N, Yancopoulos GD, Ruddy M, Bansal A (2020) Efficacy and safety of dupilumab in adolescents with uncontrolled moderate to severe atopic dermatitis: A phase 3 randomized clinical trial. JAMA Dermatol 156:44–56. https://doi.org/10.1001/jamadermatol.2019.3336

Regeneron - Internal
